# Effectiveness and cost-effectiveness of MicroShunt implantation versus standard trabeculectomy for open-angle glaucoma (a SIGHT study): study protocol of a multicentre randomised controlled trial

**DOI:** 10.1186/s12886-022-02734-y

**Published:** 2023-01-31

**Authors:** Lotte M. J. Scheres, Frank J. H. M. van den Biggelaar, Bjorn Winkens, Stefani Kujovic-Aleksov, Rogier P. H. M. Müskens, Peter W. T. de Waard, Ronald M. P. C. de Crom, Paul J. G. Ernest, Benjamin J. Pijl, Wishal D. Ramdas, Laurentius J. van Rijn, Annelie Tan, Carmen D. Dirksen, Henny J. M. Beckers

**Affiliations:** 1 University Eye Clinic Maastricht, Maastricht University Medical Centre, P. Debyelaan 25, 6229 HX, Maastricht, The Netherlands; 2 Department of Methodology and Statistics, Faculty of Health, Medicine and Life Sciences (FHML), Care and Public Health Research Institute (CAPHRI), Maastricht University, Maastricht, The Netherlands; 3 Department of Ophthalmology, Zuyderland Medical Centre, Heerlen, The Netherlands; 4 Department of Ophthalmology, University Medical Centre Groningen, Groningen, The Netherlands; 5 Department of Glaucoma, Rotterdam Eye Hospital, Rotterdam, The Netherlands; 6 Department of Ophthalmology, Bravis Hospital, Bergen op Zoom, The Netherlands; 7 Department of Ophthalmology, Deventer Hospital, Deventer, The Netherlands; 8 Department of Ophthalmology, Erasmus Medical Centre, Rotterdam, The Netherlands; 9 Department of Ophthalmology, Amsterdam University Medical Centre, Amsterdam, The Netherlands; 10 Department of Ophthalmology, Leiden University Medical Centre, Leiden, The Netherlands; 11 Department of Clinical Epidemiology and Medical Technology Assessment, CAPHRI School for Public Health and Primary Care, Maastricht University Medical Centre, Maastricht, The Netherlands

**Keywords:** MicroShunt, Trabeculectomy, Novel bleb-forming glaucoma surgery, Glaucoma, Intraocular pressure, Patient reported outcome measures, Randomized controlled trial, Cost-effectiveness, Budget impact

## Abstract

**Background:**

Trabeculectomy is the “gold standard” initial surgical procedure for open-angle glaucoma worldwide. During the last decade, the introduction of less invasive procedures, including new bleb-forming surgery such as the MicroShunt, has altered the approach of glaucoma management. At present, there is insufficient evidence comparing the effectiveness between these procedures nor versus trabeculectomy. Furthermore, there is no data available on patient impact and cost-effectiveness. This study aims to address this gap in evidence and establish whether MicroShunt implantation is non-inferior compared to trabeculectomy with regard to effectiveness and whether it is cost-effective.

**Methods:**

A multicentre, non-inferiority, randomised controlled trial (RCT) studying open-angle glaucoma with an indication for surgery will be conducted. Patients with previous ocular surgery except for phacoemulsification are excluded, as are patients with ocular comorbidity compromising the visual field or requiring a combined procedure. After informed consent is obtained, patients will be randomly allocated to the intervention, a PRESERFLO™ MicroShunt implantation, or the control group, trabeculectomy, using block randomisation (blocks of 2, 4 or 6 patients). In total, 124 patients will be randomised in a 1:1 ratio, stratified by centre. The primary endpoint will be intraocular pressure (IOP) one year after surgery. Secondary outcomes include IOP-lowering medication use, treatment failure, visual acuity, visual field progression, additional interventions, adverse events, patient-reported outcome measures (PROMs), and cost-effectiveness. Study outcomes will be measured up to 12 months postoperatively.

**Discussion:**

This study protocol describes the design of a multicentre non-inferiority randomised controlled trial. To this date, cost-effectiveness studies evaluating the MicroShunt have not been undertaken. This multicentre RCT will provide more insight into whether MicroShunt implantation is non-inferior compared to standard trabeculectomy regarding postoperative IOP and whether MicroShunt implantation is cost-effective.

**Trial registration:**

ClinicalTrials.gov, Identifier: NCT03931564, Registered 30 April 2019.

## Background

Glaucoma is characterized by progressive optic nerve degeneration and visual field loss. Ten percent of glaucoma patients will become blind in both eyes or encounter severe visual field loss in both eyes at one point in their life [1]. There is no causative treatment; thus, treatment is aimed at lowering intraocular pressure (IOP) to a target level to prohibit further damage [2]. Treatment often starts with topical medication and/or laser treatment. For patients with more advanced and worsening glaucoma, surgery is indicated.

Worldwide, trabeculectomy remains the “gold standard” initial surgical procedure for open-angle glaucoma. It creates a guarded fistula, redirecting aqueous humour into the subconjunctival space, creating a filtering bleb. It is highly effective for lowering IOP, and its safety profile has been increased due to ample experience with the procedure [3]. However, postoperative care is of utmost importance in achieving optimal bleb function and, consequently, successful results. Furthermore, trabeculectomy often causes discomfort and loss of visual acuity, taking weeks to months before vision is restored, and mild to serious complications can occur, such as hypotony, cataract formation, and bleb infection, which may require additional surgery [4, 5].

During the past decade, the introduction of minimally and less invasive glaucoma surgery has led to a substantially altered approach of glaucoma management [6]. These procedures are promising for filling the gap between medical therapy and traditional surgeries that are burdensome to the patient and have a known risk of complications. The surgeries are faster and easier to perform, causing less tissue trauma, faster patient recovery, fewer postoperative visits, and possibly less impact on visual function and quality of life, all whilst retaining the possibility of performing additional glaucoma surgery [7, 8].

New bleb-forming devices using a subconjunctival drainage approach may lead to a similar IOP reduction compared to trabeculectomy, reaching a target in the low teens. A recently developed bleb-forming device is the PRESERFLO™ MicroShunt (Santen, Osaka, Japan) (MicroShunt) [9]. The MicroShunt is an 8.5 mm long stent made of a novel polymer, ‘SIBS’. This material is biostable, thermoplastic, and provokes less inflammation in comparison to other commonly used materials. Published studies have shown encouraging results, although the current peer-reviewed literature on the MicroShunt is still mainly limited to non-comparative case series and feasibility studies [10–14]. A Cochrane systematic review from 2018 published by King and colleagues [15], investigating the effects of subconjunctival draining glaucoma devices for open-angle glaucoma, stated the need for properly designed randomised controlled trials (RCT). A recently published commercially driven clinical Food and Drug Administration (FDA) trial compared the safety and efficacy of the MicroShunt and trabeculectomy in primary open-angle glaucoma (POAG) patients [16]. However, this study only addresses the efficacy of the two treatments and does not consider the impact on patients or economic consequences.

Cost-effectiveness studies evaluating the MicroShunt have not yet been undertaken. An increase in the number of glaucoma surgeries is expected due to the aging of the population and because patients will have access to these new options at an earlier stage in their treatment algorithm [17]. This increase warrants the need for surgical options that are cost-effective compared to current treatments. MicroShunt implantation is currently not considered in accordance with standards of science and practice and is not reimbursed by healthcare insurers in the Netherlands. Hence, a societal cost-effectiveness analysis needs to be undertaken to further elucidate its position in the glaucoma treatment algorithm in the Netherlands.

This investigator-initiated multicentre randomised controlled study aims to evaluate the effectiveness and cost-effectiveness of the MicroShunt compared to trabeculectomy with mitomycin C (MMC) in the Netherlands in open-angle glaucoma patients to test the hypothesis that MicroShunt is non-inferior to trabeculectomy in terms of effectiveness and superior in terms of cost-effectiveness.

## Methods/design

### Objective

The aim of this study is to evaluate whether MicroShunt implantation is non-inferior regarding effectiveness, where effectiveness is assessed using the IOP after 12 months of follow-up, as compared to trabeculectomy in open-angle glaucoma patients. In addition, IOP-lowering medication use, treatment failure, visual acuity, visual field progression, additional interventions, adverse events, patient-reported outcome measures (PROMs), and cost-effectiveness are evaluated.

### Study design

**Fig. 1:**
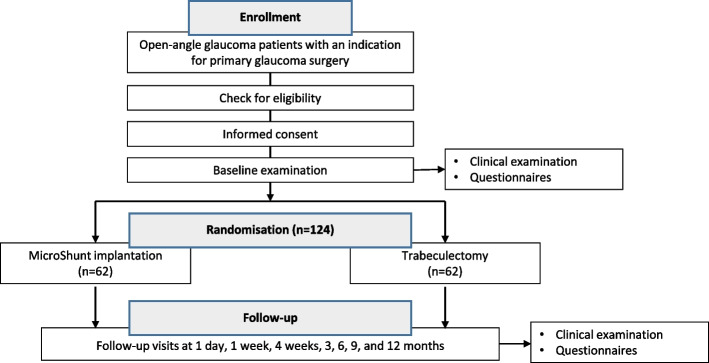
Study design

A multicentre, non-inferiority, single-blinded, randomised controlled clinical trial will be performed at outpatient ophthalmology clinics in the Netherlands. The follow-up duration will be one year. The study design is presented in Fig. 1.

### Participating hospitals

Hospitals with glaucoma surgeons that regularly perform trabeculectomy can participate in this trial. Five academic centres (Amsterdam University Medical Center; Erasmus Medical Center; Leiden University Medical Center; Maastricht University Medical Center+; University Medical Center Groningen), and four non-academic centres (Bravis Hospital, Bergen op Zoom; Deventer Hospital; The Rotterdam Eye Hospital; Zuyderland Medical Center, Heerlen) will participate. Surgeons will be trained for the MicroShunt procedures before participating in the study to minimize a learning curve. Before the start of the trial, a masterclass will be organised to reach a consensus on the details of the study methods and the surgical technique.

### Study population

**Fig. 2:**
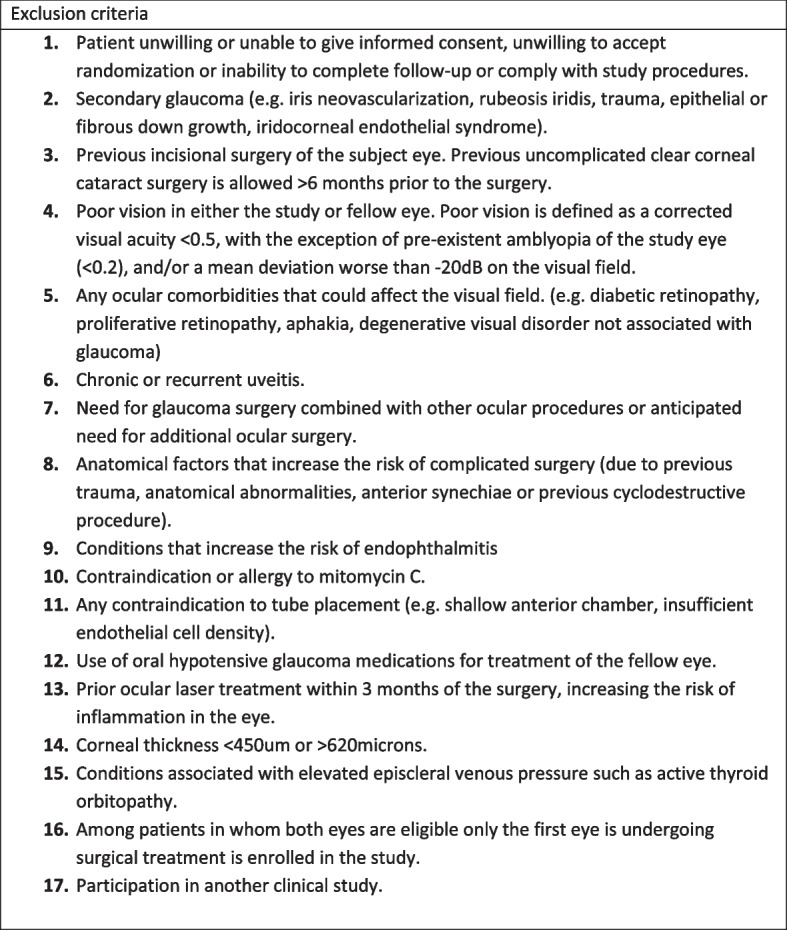
Exclusion criteria

Caucasian patients between 18 and 80 years old with uncontrolled open-angle glaucoma on maximum tolerated medical therapy and/or progression on the visual field with an indication for primary glaucoma surgery will be suitable for inclusion. The exclusion criteria are listed in Fig. 2.

### Recruitment, consent, and randomisation

Patients meeting the eligibility criteria will be counselled about the study by their ophthalmologist. Subsequently, a local researcher will provide them with verbal and written information. Patients who agree to participate in the study must provide written informed consent before randomisation is performed.

The certified electronic data capture tool Castor EDC (Castor Electronic Data Capture, version 2022.2.2.2, Ciwit BV, Amsterdam, the Netherlands) will be used for randomisation, sending out questionnaires, and data collection [18]. The computer will assign each patient a unique code, consisting of a centre-specific prefix and a serial number. The subject code will then be allocated to either MicroShunt implantation (intervention) or trabeculectomy (control) in a 1:1 ratio through block randomisation, stratified according to centres, and using variable block sizes of 2, 4, and 6. Study participants will be blinded to the intervention for one year. Due to the nature of the intervention, it is impossible to blind the treating physician.

### Interventions

The intervention consists of MicroShunt implantation augmented with MMC application. It comes in a package including the surgical instruments required to implant the device. A detailed description of the procedure can be found elsewhere [19, 20]. To summarize, a fornix-based conjunctival/Tenon’s dissection will be made, and a deep sub-Tenon’s pocket will be formed. After application of MMC, the MicroShunt will be implanted into the anterior chamber through a 25-gauge scleral needle track, and the Tenon’s and conjunctiva will be sutured in a watertight fashion.

The intervention will be compared to the usual care, which is a fornix-based trabeculectomy augmented with MMC application, according to the surgeon’s preferred technique. Both procedures may be performed under local anaesthesia or general anaesthesia conformable to local conditions in the participating centre. After surgery, topical anti-inflammatory therapy will be commenced and gradually tapered off according to bleb formation. IOP-lowering medication may be (re)introduced according to the discretion of the treating ophthalmologist. In the event of an impending bleb failure, further surgical interventions may be performed.

### Outcome measures

The primary outcome will be the level of IOP one year after surgery. IOP will be measured by Goldmann tonometry in mmHg.

Secondary outcomes will include:

1. IOP-lowering medication use: the number of active substances.
2. Failure rate: failure is defined as an IOP > 21 mmHg and ≤ 5 mmHg or less than 20% reduction relative to baseline IOP at two consecutive follow-up visits after three months. Reoperation will also be defined as failure. Eyes that have not failed and are not on supplemental medical therapy are considered complete successes. Eyes that have not failed but require supplemental medical therapy are defined as qualified successes [21].
3. Visual acuity: corrected distance visual acuity (CDVA) in the logarithm of the mean angle of resolution (logMAR).
4. Visual field progression: mean deviation on the Humphrey Field Analyser.
5. Safety: the number and types of adverse events and the number and types of additional surgical interventions.
6. PROMs, including vision-related quality of life and general health-related quality of life: a. Condition-specific quality of life and patient satisfaction will be measured using the National Eye Institute Visual Function Questionnaire-25 (NEI-VFQ-25) [22] and the Glaucoma Quality of Life-15 (GQL-15) [23]. b. Health-related quality of life (HRQL) will be measured using two questionnaires: EuroQol’s EQ-5D-5L [24] and the Health Utilities Index Mark-3 (HUI-3) [25].
7. Incremental cost-effectiveness ratios (ICERs): these will be expressed as 1) incremental societal costs per quality-adjusted life year (QALY) gained, 2) incremental healthcare costs per clinically improved patient on the NEI VFQ-25 questionnaire, 3) the incremental healthcare costs per clinically improved patient on the GQL-15 questionnaire, and 4) the incremental healthcare costs per patient with clinically lowered IOP.
8. Budget impact will be reported as difference in costs. Various scenarios will be compared to investigate the impact of various levels of implementation.

### Study measurements

An overview of the study measurements per visit is presented in Table 1. Enrolled patients complete follow-up visits at one day, one week, four weeks, three months, six months, and one year after surgery. Time windows are based on the proposed time windows of the guidelines on design and reporting of the World Glaucoma Association (WGA) [21].

**Table 1:** Follow-up measurements at each time point

Assessment/ procedure	Baseline	Procedure	Follow-up examination						
			1 day	1 week	4 weeks	3 months	6 months	9 months	12 months
Check for in−/exclusion criteria	x								
Informed Consent	x								
Medical History/ Demographics	x								
Ocular medication assessment	x	x	x	x	x	x	x	x	x
Manifest refraction	x								x
Autorefraction	x			x	x	x	x	x	x
CDVA	x			x	x	x	x	x	x
Visual field	x								x
Slit beam examination	x		x	x	x	x	x	x	x
Slit lamp photo				x	x	x			x
IOP	x		x	x	x	x	x	x	x
Pachymetry	x								x
Endothelial cell density	x								x
Eye motility	x				x				x
Gonioscopy	x								
Fundoscopy	x								
Questionnaires									
NEI VFQ-25	x				x	x	x		x
GQL-15	x				x	x	x		x
EQ-5D-5L	x				x	x	x		x
HUI-3	x				x	x	x		x
Standardized cost questionnaire	x					x	x		x
Safety									
Complications		x	x	x	x	x	x	x	x
Additional interventions		x	x	x	x	x	x	x	x

Abbreviations: *CDVA* Corrected distance visual acuity, *IOP* Intraocular pressure

## Statistical analysis

### Sample size

The sample size is based on the primary outcome, IOP, a numerical response variable, to detect non-inferiority of an experimental group compared to a control group at 12 months of follow-up. A couple of studies have been published on the efficacy of the experimental group. Based on observed means of postoperative IOP from these studies [11, 26, 27], a mean IOP for the experimental group of 12 mmHg was chosen. The non-inferiority margin (control minus intervention) of − 2.5 mmHg is chosen based on previous trials investigating glaucoma surgery [26, 28, 29]. Assuming this non-inferiority margin of − 2.5 mmHg, a within-group standard deviation (SD) of 4.4, and an expected effect (control minus intervention) of 0.4 [5], 49 patients per group will be required to show non-inferiority with 90% power and a one-sided significance level alpha of 0.025. Accounting for 20% loss-to-follow up, we will need to include 62 patients per group, i.e., 124 patients in total.

As for secondary outcome parameters, this number of patients should be sufficient to detect relevant differences in CDVA and postoperative intervention and complication rates. A 0.13 logMAR difference between the two groups (within group SD = 0.2) can be detected with 90% power and a two-sided alpha of 0.05. In the primary tube versus trabeculectomy study [5], the postoperative intervention and complication rate in the control group was 63 and 41%, respectively. With the planned sample size for our study (124 patients in total with 20% dropout), we can detect a difference of 32% (63% in the control group versus up to 31% in the intervention group) for postoperative interventions and 28% (41% in the control group versus 13% in the intervention group) for postoperative complications, with 90% power and a one-sided significance level alpha of 0.025.

### Data-analysis

A single main analysis will be performed at the end of the trial when all follow-up visits have been completed.

The data analysis will be performed according to the intention-to-treat principle. Additionally, a per-protocol analysis will be performed, as is recommended for non-inferiority trials [30]. The per-protocol population includes all randomized patients who complete treatment without major protocol deviations. The details of the number of eligible patients for the trial, the number of consenting, and the number of randomised patients will be shown graphically as a CONSORT diagram [31], to describe the course of patients throughout the trial.

Baseline characteristics will be presented as means with standard deviation and 95% confidence intervals, as median and interquartile range (IQR), or as frequencies (with percentages), as appropriate. For the primary outcome, IOP, a linear mixed model analysis will be used since it accounts for the stratification variable (centre), baseline differences, uses all available data, corrects for correlation between repeated measures, and assumes missing data to be missing at random (MAR). A similar model will be used for the secondary outcomes that are also repeatedly measured. Different options will be considered for the covariance structure of the repeated measures as well as the random part of the model, and the one with the lowest Bayesian Information Criterion (BIC) will be chosen. To determine the success rates, Kaplan-Meier survival methods as well as Cox regression analysis will be used.

### Economic evaluation

A trial-based cost-effectiveness analysis will be conducted from a societal and a healthcare perspective over a 12-month time horizon. A detailed and complete analysis of the costs of each patient included in the study up to 12 months from a healthcare and societal perspective will be performed. Resource use will be collected by means of the case report form (CRF) regarding both procedures, (pre- and postoperative) visits, and treatment of adverse events. Information on resource use outside of the hospital (e.g., productivity losses, homecare) will be collected by means of a short cost questionnaire. Valuation of resource use will be performed in accordance with the Dutch guideline for cost analysis [32] using either reference prices or local cost prices provided by the Maastricht University Medical Center+, whichever is deemed more appropriate.

QALYs will be calculated based on EuroQol’s EQ-5D-5L using the Dutch tariffs [24, 33]. Additionally, the HUI-3 will be used because it is the only generic preference-based HRQL questionnaire that includes questions about vision [25].

ICERs will be calculated for the subtypes described earlier in this paper and are calculated by dividing the cost difference between the groups by the difference in effects. To estimate uncertainty in the point estimates of the ICERs, bootstrap cost-effect pairs will be plotted on cost-effectiveness planes. The probability that the MicroShunt is cost-effective will be estimated using cost-effectiveness acceptability curves for a range of threshold values. Additional sensitivity analyses will be performed to investigate the impact of varying input variables.

### Budget impact analysis

Alongside the cost-effectiveness analysis, a budget impact analysis (BIA) will be performed to evaluate the impact of implementation of the MicroShunt on the Dutch healthcare budget compared to standard trabeculectomy. The BIA addresses the financial stream of consequences related to the implementation of the MicroShunt to assess affordability and will be performed in accordance with the Dutch guidelines for economic evaluations and the International Society for Pharmacoeconomics and Outcomes Research (ISPOR) guidelines [33, 34]. Costs will be calculated using similar methods to those described in the cost-effectiveness analysis section. Prices will be corrected to the price levels of each specific budget period (i.e., one calendar year), and costs will not be discounted. A simple decision analytic model will be built using Microsoft Excel (Microsoft Corporation, Redmond, Washington, United States, version 2016 or higher). The analysis will use the perspective of the budget holder, complemented with additional perspectives such as healthcare providers and insurers. A time horizon of five years will be used to account for the fact that implementation will take place gradually. It will be assumed that the difference in costs will be related entirely to the substitution of trabeculectomy by MicroShunt implantation. Various scenarios will be compared to investigate various levels of implementation (i.e., 25, 50, 75, and 100% of eligible patients). Furthermore, scenarios will be modelled in which the timeline of implementation in 100% of the hospitals varies from direct implementation to implementation in five years. Sensitivity analyses will be performed to test the robustness of the analysis.

### Data management and monitoring

Since this study investigates an approved intervention within its indication of use, a Data Safety Monitoring Board will not be installed, and an interim analysis will not be conducted.

Personal data will be handled confidentially. Data will be collected in the online CRF by a member of the study team of each study centre. The dataset is coded, and the local research team will have access to the key. All data will be stored and analysed using the unique study code. After completion of the study, the data will be archived for 15 years. Monitoring will be performed by the Clinical Trial Center Maastricht (CTCM), an academic research organisation affiliated with the sponsor, in order to protect patient rights and the accuracy of the reported trial data. Site monitoring visits will take place to assess the adherence to the study protocol and the performance of the participating sites. Qualified and independent monitors will have access to the data and source documents.

### Safety monitoring

Adverse events (AE) are defined as any undesirable experience occurring to a subject during the study. All AEs related to the study eye, predefined by the WGA [21], reported spontaneously by the subject or observed by the investigator or his staff will be recorded unless the event is of negligible impact and, in addition, has no connection to the study anywise (e.g., the common cold). All serious adverse events (SAE) will be reported by the local investigator to the principal investigator. The principal investigator will report a SAE to the ethics committee of Maastricht University Medical Centre+ and Maastricht University (azM/UM) within a period of maximum 15 days after the first knowledge of the serious adverse event. If SAEs occur that are unrelated to the intervention, these SAEs will be reported in line listing in the annual progress report.

## Discussion

The introduction of less invasive surgical methods, including new bleb-forming surgeries such as the MicroShunt, has altered the approach of glaucoma treatment worldwide. The current lack of consensus on the use of these procedures relates to the lack of evidence. Many studies have evaluated trabeculectomy with antifibrotic agents and, therefore, it is strongly recommended as an initial surgical treatment for open-angle glaucoma. At present, there is not sufficient evidence comparing the effectiveness between these procedures nor versus trabeculectomy. Furthermore, the available data remains limited, and currently, it remains unclear whether less invasive procedures are cost-effective [35]. The current study aims to address this current gap in evidence.

A recently published RCT that directly compared trabeculectomy with MicroShunt implantation reported lower IOP levels in the trabeculectomy group after one year [16]. However, this is a commercially driven FDA trial. Its study population was restricted to POAG patients, and baseline characteristics were unequal with regards to race. Additionally, the authors discuss a possible learning curve in the participating surgeons concerning the intervention group, and MMC application was limited to a concentration of 0.2 mg/ml applied for two minutes. Furthermore, this study does not entail an economic evaluation. Our proposed study is the first trial that includes all open-angle glaucoma patients, such as POAG, pseudoexfoliation syndrome, and pigment dispersion syndrome. It is not limited to the use of a specific MMC concentration to augment the procedure, includes only experienced glaucoma surgeons in the Netherlands, and investigates patient-reported outcomes, quality of life, and cost-effectiveness. To our knowledge, there is currently no further evidence available comparing MicroShunt implantation or other less invasive methods to trabeculectomy in a RCT.

The current study is the first multicentre RCT that aims to establish whether, for open-angle glaucoma patients without previous surgery (except for clear corneal phacoemulsification), MicroShunt implantation is non-inferior in comparison to trabeculectomy with regard to effectiveness, and whether it is cost-effective. We anticipate that this study will provide valuable information for decision-making and potentially alter the current approach to glaucoma treatment in the Netherlands.

## Trial status

The trial has started recruitment on the 10th of March 2020, and is expected to be completed by June 2023. The latest version of the study protocol is version 7.0 (date: 26-04-2022).

## Data Availability

Not applicable.

## Abbreviations

AE: Adverse Event
BIA: Budget Impact Analysis
BIC: Bayesian Information Criterion
CDVA: Corrected Distance Visual Acuity
CRF: Case Report Form
CTCM: Clinical Trial Center Maastricht
EDC: Electronic Data Capture
FDA: Food and Drug Administration
GQL-15: Glaucoma Quality of Life-15
HRQL: Health-related Quality of Life
HUI-3: Health Utility Index Mark 3
ICER(s): Incremental cost-effectiveness ratio(s)
IOP: Intraocular Pressure
IQR: Interquartile Range
ISPOR: International Society for Pharmacoeconomics and Outcomes Research
LogMAR: Logarithm of the Minimum Angle of Resolution
MAR: Missing at Random
MMC: Mitomycin C
NEI VFQ-25: National Eye Institute Visual Function Questionnaire-25
PROM(s): Patient Reported Outcome Measure(s)
QALY(s): Quality-Adjusted Life Year(s)
RCT: Randomised Controlled Trial
SAE: Serious Adverse Event
SD: Standard Deviation
WGA: World Glaucoma Association
